# Effects of prolonged type 2 diabetes on changes in peripapillary retinal nerve fiber layer thickness in diabetic eyes without clinical diabetic retinopathy

**DOI:** 10.1038/s41598-021-86306-y

**Published:** 2021-03-24

**Authors:** Min-Woo Lee, Hyung-Bin Lim, Min-Su Kim, Gi-Seok Park, Ki-Yup Nam, Young-Hoon Lee, Jung-Yeul Kim

**Affiliations:** 1 Department of Ophthalmology, Konyang University College of Medicine, Daejeon, Republic of Korea; 2 Department of Ophthalmology, Chungnam National University College of Medicine, #640 Daesa-dong, Jung-gu, Daejeon, 301-721 Republic of Korea; 3 Department of Ophthalmology, Chungnam National University Sejong Hospital, Sejong, Republic of Korea

**Keywords:** Medical research, Pathogenesis

## Abstract

To identify the effects of prolonged type 2 diabetes (T2DM) on changes in peripapillary retinal nerve fiber layer (pRNFL) thickness in patients without clinical diabetic retinopathy. Subjects were divided into two groups: controls and patients with T2DM (DM group). After the initial visits, the pRNFL thicknesses were measured three more times at 1-year intervals. Subgroup analyses were performed in patients with T2DM duration ≥ 10 years. The mean pRNFL thickness at each visit was 95.8 ± 8.1, 95.4 ± 8.3, 94.9 ± 8.1, and 94.5 ± 8.3 μm in the control group (*P* = 0.138) (n = 55); and 93.4 ± 9.1, 92.1 ± 9.3, 90.9 ± 9.3, and 89.5 ± 9.2 μm in the DM group (*P* < 0.001) (n = 85). The estimated rate of reduction in mean pRNFL thickness was − 0.45 μm/year in the control group and − 1.34 μm/year in the DM group, respectively. In the DM group, the BCVA and HbA1c (both *P* = 0.001) were significant factors associated with pRNFL reduction. In patients with T2DM duration ≥ 10 years, the estimated pRNFL reduction rate was − 1.61 μm/year, and hypertension was a significant factor affecting the pRNFL reduction (*P* = 0.046). We confirmed rapid pRNFL reduction over time in T2DM, and the reduction rate was higher in patients with T2DM ≥ 10 years. Additionally, BCVA and HbA1c levels were significantly associated with the change in pRNFL thickness in T2DM patients.

## Introduction

Type 2 diabetes (T2DM) constitutes a global health problem and induces macrovascular and microvascular complications causing more than 2 million deaths every year^1^. Moreover, its prevalence has increased from 108 million in 1980 to 422 million in 2014, and is expected to increase to 629 million by 2045 because of lifestyle changes^2–4^. This increase in T2DM is associated with an increase in diabetic retinopathy (DR)^5^. DR is one of the most common complications of T2DM and is the leading cause of preventable blindness among working-age subjects in most developed countries^5,6^. Even before clinical DR emerges, many studies have reported functional and anatomical damages on the retina by T2DM.

Diabetic retinal neurodegeneration (DRN) is one of the representative damages on the retina by T2DM showing before clinical DR. Metabolic pathways triggered by hyperglycemia such as the polyol and hexosamine pathways, the de novo synthesis of diacylglycerol-protein kinase C, and the production of free radicals are closely associated with DRN, which causes the apoptosis of retinal ganglion cells^7,8^. Such retinal ganglion cell loss would result in a reduction in the thickness of the retinal nerve fiber layer (RNFL). Vujosevic et al.^9^ reported early changes in the peripapillary microvasculature that correlated with peripapillary retinal nerve fiber layer (pRNFL) thinning in T2DM patients without DR. Lim et al.^10^ found that T2DM was associated with accelerated pRNFL loss regardless of whether or not DR progression, suggesting that DRN may proceed the microvascular abnormalities associated with DR progression. Additionally, they reported that the T2DM duration was associated with longitudinal changes in pRNFL thickness. However, few studies have explored the severity of DRN according to T2DM duration.

The purpose of this study was to identify the effects of prolonged T2DM on longitudinal changes in pRNFL by comparing pRNFL thickness of normal individuals and DM patients.

## Methods

### Patients

This prospective, longitudinal, observational study adhered to the tenets of the Declaration of Helsinki and was approved by the Institutional Board and Ethics Committee of Chungnam National University Hospital, Daejeon, Republic of Korea. Patients with T2DM were enrolled in the ‘Changes in the inner retina in diabetes patients with or without diabetic retinopathy’, which is an ongoing prospective study. The control group included patients diagnosed with a unilateral epiretinal membrane, macular hole, or intraocular lens dislocation; all of the fellow eyes without any ophthalmic disease. Written informed consent was obtained from all subjects.

Subjects were divided into two groups: controls and patients with T2DM (DM group). Additionally, we performer subgroup analyses for T2DM patients with DM duration ≥ 10 years in the DM group. All patients underwent complete ophthalmic examinations including measurements of best-corrected visual acuity (BCVA), intraocular pressure, the spherical equivalent, and axial length; a detailed fundus examination; and spectral-domain optical coherence tomography (OCT). After the initial visit, three further examinations were performed times at 1-year intervals. The exclusion criteria were a history of any systemic disease other than T2DM and hypertension, any ophthalmic diseases such as glaucoma, retinal diseases, or neuro-ophthalmic diseases, an axial length ≥ 26.0 mm, any prior intraocular surgery except cataract extraction, a BCVA < 20/40, and intraocular pressure > 21 mm Hg as previous studies^10^. We also excluded patients exhibiting clinical evidence of DR such as retinal hemorrhages or microaneurysm using fundus photo and dilated fundus examination. One eye was randomly selected in patients in whom both eyes met the inclusion criteria.

### OCT measurements

OCT measurements were performed by a skilled examiner using a Cirrus HD OCT (version 10.0; Carl Zeiss Meditec, Dublin, CA, USA). The pRNFL thicknesses were measured from optic disc cube scans. A 200 × 200 scan optic cube (measured in pixels) scanning protocol was used to image the optic disc and pRNFL over a 6 × 6-mm region of the optic nerve head. Images of signal strength < 7, any motion artifact, involuntary saccade, obvious decentration misalignment, or algorithm segmentation failure identified by the auto segmentation error on B-scan images were excluded as previous studies^10^.

### Statistical analyses

Demographic characteristics and ocular parameters were compared via the independent t-test and the chi-squared test. Repeated-measures ANOVA was used to analyze longitudinal changes in the pRNFL thicknesses in each group. Linear mixed models were performed to reveal the reduction rate of the pRNFL in each group and differences in such reduction over time among the groups. The variables that may have any effect on the results and can be assessed in the clinical situation such as age, sex, the BCVA, the spherical equivalent, intraocular pressure, axial length, follow-up duration, baseline central macular thickness, baseline ganglion cell-inner plexiform layer (GC-IPL) thickness, baseline pRNFL thickness, HbA1c level, hypertension status, and the interaction between follow-up duration and group were included as fixed effects, and the eye levels were included as the random intercept. Diagnosis-stratified mixed models were fitted to identify factors associated with longitudinal changes in mean pRNFL thickness in each group.

## Results

### Demographics

A total of 140 eyes were included: 55 in the control group, and 85 in the DM group. The mean age in each group was 61.1 ± 8.7 and 61.1 ± 8.2, respectively (*P* = 0.998) (Table 1). The sex, BCVA, spherical equivalent, axial length, and optic nerve head parameters did not differ significantly among the groups. The DM duration was 9.78 ± 6.48, and the HbA1C level was 7.00 ± 0.97 in the DM group.

**Table 1:** Demographics and clinical characteristics.

	Controls (n = 55)	DM group (n = 85)	*P* value
Age (mean ± SD, years)	61.1 ± 8.7	61.1 ± 8.2	0.998
Sex (male, %)	28 (50.9%)	41 (48.2%)	0.863
BCVA (mean ± SD)	− 0.016 ± 0.057	0.008 ± 0.084	0.057
SE (mean ± SD, diopters)	0.11 ± 1.31	− 0.316 ± 1.63	0.115
IOP (mean ± SD, mmHg)	14.6 ± 2.7	15.4 ± 3.0	0.116
Axial length (mean ± SD, mm)	23.7 ± 1.0	23.9 ± 0.9	0.194
Hypertension (n, %)	45 (81.8%)	58 (68.2%)	0.081
DM duration (mean ± SD, years)	0	9.78 ± 6.48	N/A
HbA1C (mean ± SD, %)	N/A	7.00 ± 0.97	N/A
ONH rim area (mean ± SD, mm^2^)	1.32 ± 0.26	1.23 ± 0.24	0.065
Disc area (mean ± SD, mm^2^)	1.90 ± 0.29	1.96 ± 0.38	0.339
Cup-disc ratio (mean ± SD)	0.53 ± 0.14	0.56 ± 0.18	0.218
CMT (mean ± SD, μm)	252.9 ± 18.6	253.3 ± 21.6	0.915
GC-IPL thickness (mean ± SD, μm)	83.6 ± 6.5	80.9 ± 7.7	**0.031**

Values in boldface (*P* < 0.05) are statistically significant.

*SD* standard deviation, *BCVA* best-corrected visual acuity, *SE* spherical equivalent, *IOP *intraocular pressure, *DM* diabetes, *ONH* optic nerve head, *CMT* central macular thickness, *GC-IPL* ganglion cell-inner plexiform layer.

### Peripapillary retinal nerve fiber layer thicknesses

**Figure 1:**
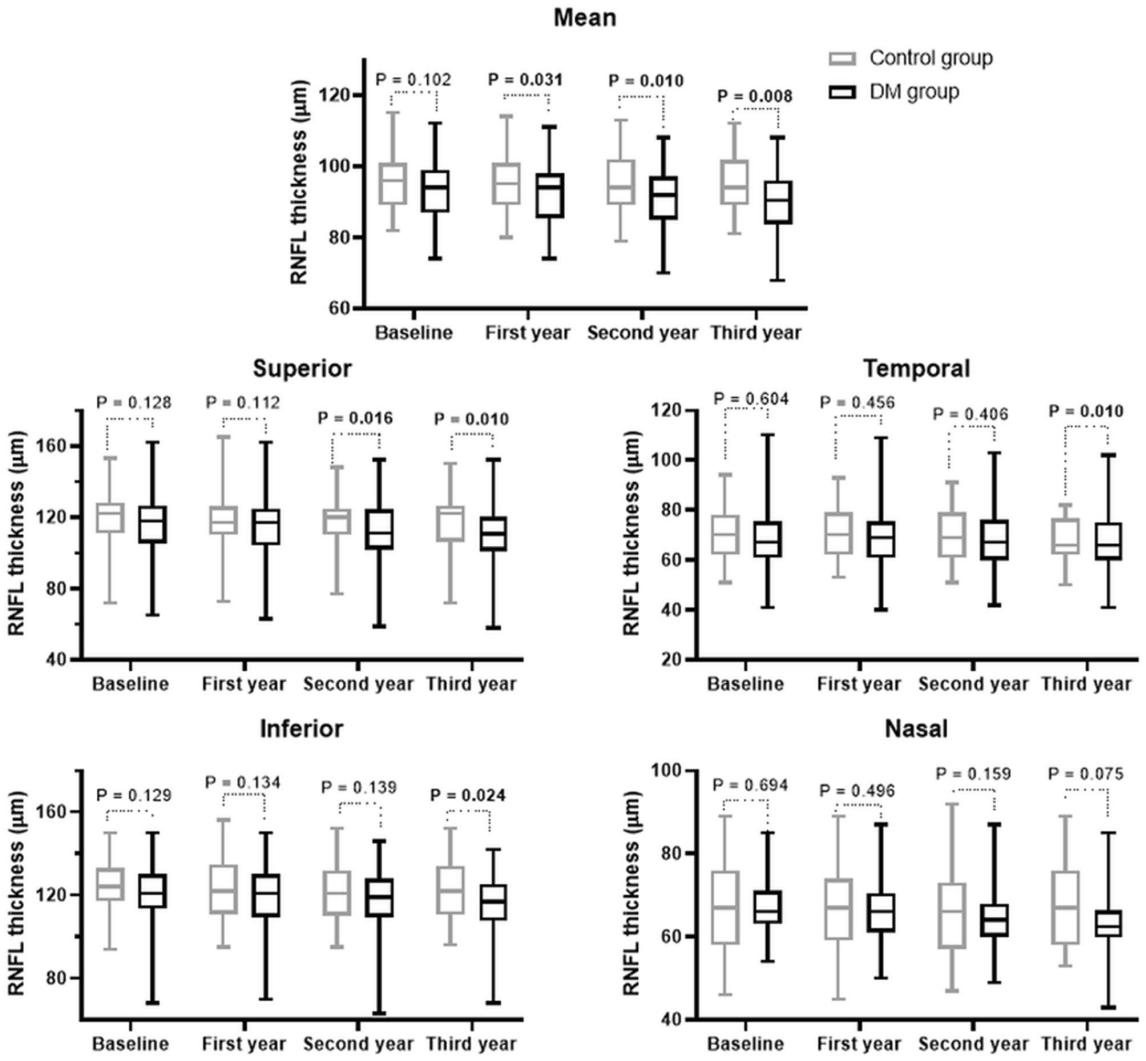
Mean and sectoral peripapillary retinal nerve fiber layer (RNFL) thicknesses according to visit. Boxes are 25–75% quartiles, lines in the boxes are the medians, and the whiskers indicate variability (minimum and maximum values).

The mean pRNFL thickness in the control group at each visit was 95.8 ± 8.1, 95.4 ± 8.3, 94.9 ± 8.1, and 94.5 ± 8.3 μm, which showed a continuous reduction, but was not significant statistically (*P* = 0.138) (Fig. 1). The pRNFL thicknesses of the four segments also showed a decreasing trend, which did not differ significantly. In the DM group, the mean pRNFL thicknesses at each visit were 93.4 ± 9.1, 92.1 ± 9.3, 90.9 ± 9.3, and 89.5 ± 9.2 μm, which showed a significant reduction over time (*P* < 0.001). The superior and inferior segment thicknesses also decreased significantly over time (both *P* < 0.001) (Supplementary table).

### Reduction rates of pRNFL thickness

The estimated mean reduction rates of pRNFL thickness were − 0.45 μm/year ear in the control group and − 1.34 μm/year in the DM group (Table 2). The thicknesses of all four segments decreased significantly over time in the DM group. The reduction of mean pRNFL thickness over time was significantly different between the control and DM groups (*P* < 0.001).

**Table 2:** Estimated mean rates of reduction in peripapillary retinal nerve fiber layer from the linear mixed model.

	Control		DM group		*P *value^a^
	Reduction (95% CI), μm/year	*P* value	Reduction (95% CI), μm/year	*P* value	
Mean	− 0.45 (− 0.88 to − 0.02)	**0.040**	− 1.34 (− 1.56 to − 1.11)	**< 0.001**	**< 0.001**
**Segment**					
Superior	− 0.67 (− 1.36 to 0.03)	0.059	− 1.98 (− 2.49 to − 1.48)	**< 0.001**	**0.004**
Temporal	− 0.30 (− 0.74 to 0.14)	0.172	− 0.66 (− 0.90 to − 0.41)	**< 0.001**	0.164
Inferior	− 0.81 (− 1.67 to 0.35)	0.060	− 1.41 (− 1.83 to − 1.00)	**< 0.001**	**0.046**
Nasal	− 0.30 (− 0.88 to 0.28)	0.296	− 1.11 (− 1.40 to − 0.82)	**< 0.001**	**0.005**

Values in boldface (*P* < 0.05) are statistically significant.

^a^Calculated for interaction between groups and follow-up duration in the linear mixed model.

### Factors associated with changes in mean pRNFL thickness

Baseline pRNFL thickness was significantly associated with pRNFL reduction in both groups (Table 3). In the DM group, age (estimate = − 0.27 μm/year, *P* = 0.010), BCVA (estimate = − 51.97 μm/year, *P* = 0.001), HbA1c level (estimate = − 3.05 μm/year, *P* = 0.001), follow-up duration (estimate = − 1.28 μm/year, *P* < 0001), and baseline GC-IPL (estimate = 0.65 μm/year, *P* < 0.001) were significantly associated with pRNFL reduction.

**Table 3:** Results of linear mixed models assessing the effects of putative factors predicting changes in mean peripapillary retinal nerve fiber layer (pRNFL) thickness over time.

	Control		DM group	
	Estimate (95% CI), μm/year	*P* value	Estimate (95% CI), μm/year	*P* value
Age	− 0.10 (− 0.34 to 0.14)	0.388	− 0.27 (− 0.55 to − 0.08)	**0.010**
Sex	1.97 (− 2.17 to 6.12)	0.344	− 5.15 (− 11.20 to 0.89)	0.093
BCVA	29.84 (− 6.16 to 65.85)	0.102	− 51.97 (− 82.09 to − 21.95)	**0.001**
SE	0.37 (− 1.24 to 1.97)	0.649	− 0.28 (− 2.96 to 2.39)	0.833
IOP	0.07 (− 0.72 to 0.86)	0.859	− 0.15 (− 1.29 to 0.99)	0.792
Axial length	− 0.18 (− 2.37 to 2.02)	0.873	− 0.05 (− 3.22 to 0.13)	0.977
Hypertension	0.829 (− 4.56 to 6.22)	0.759	− 4.50 (− 9.01 to 0.02)	0.055
HbA1c	n/a	n/a	− 3.05 (− 5.12 to − 1.50)	**0.001**
DM duration	n/a	n/a	− 0.18 (− 0.33 to − 0.07)	**0.002**
Follow-up duration	− 0.49 (− 1.00 to 0.02)	0.061	− 1.28 (− 1.58 to − 0.95)	**< 0.001**
Baseline CMT	− 0.05 (− 0.16 to 0.06)	0.375	− 0.04 (− 0.10 to 0.08)	0.210
Baseline GC-IPL	0.08 (− 0.25 to 0.39)	0.675	0.65 (0.4 to 0.86)	**< 0.001**
Baseline pRNFL	0.91 (0.82 to 0.99)	**< 0.001**	0.96 (0.92 to 1.04)	**< 0.001**

*BCVA* best− corrected visual acuity, *SE* spherical equivalent, *IOP* intraocular pressure, *CMT* central macular thickness, *GC-IPL* ganglion cell-inner plexiform layer.

Values in boldface (*P* < 0.05) are statistically significant.

### Analyses of T2DM patients with DM duration ≥ 10 years

**Figure 2:**
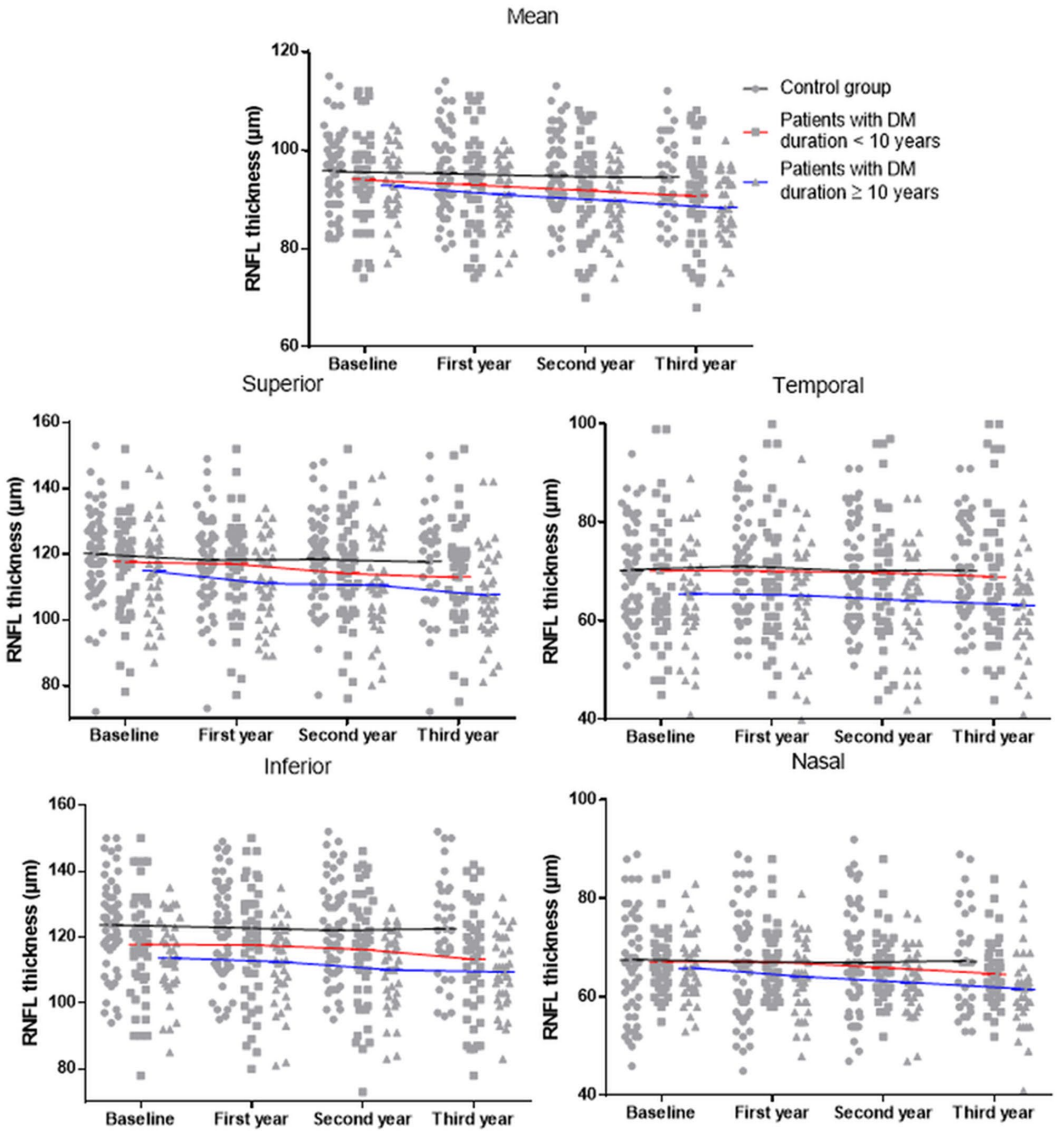
Scatterplots and line graphs of the mean and sectoral peripapillary retinal nerve fiber layer (RNFL) thicknesses at each visit in normal controls, patients with DM duration < 10 years, and patients with DM duration ≥ 10 years.

The number of patients with DM duration ≥ 10 years was 38 in the DM group. The mean pRNFL thickness at each visit was 92.8 ± 7.1, 91.1 ± 7.1, 89.8 ± 6.9, and 88.3 ± 6.9 μm, which showed a significant reduction over time (*P* < 0.001), and all four segment thicknesses also decreased significantly over time (superior, *P* = 0.001; temporal, *P* = 0.012; inferior, *P* < 0.001; nasal, *P* < 0.001) (Fig. 2).

The estimated mean reduction rate of pRNFL thickness was − 1.61 μm/year (95% CI − 1.89 to − 1.34; *P* < 0.001). Additionally, hypertension was a significant factor affecting the pRNFL change (estimate = − 4.88, *P* = 0.046). Other significant factors were similar to the result in the DM group, but age did not show a significant result (estimate = 0.05, *P* = 0.737).

## Discussion

This study identified the longitudinal reductions in pRNFL thickness in T2DM patients without DR by DRN as previous studies^7,9–11^. Such anatomical damages on the retina could be associated directly or indirectly with the functional decline of vision. Zeng et al.^12^ reported that delayed implicit time and decreased amplitude were found in T2DM patients without DR compared to the control group in electroretinography. Reduced color and contrast sensitivities, delayed dark adaptation, and compromised visual field sensitivity are also known consequences of DRN^7,11,13–16^. Although such anatomical and functional damages on the retina would occur in T2DM before emerging clinical DR, current treatments are considered at late stages of DR when vision has been irreversibly affected. Therefore, more efficient and preventive treatment strategies are required at earlier stages, based on improved understanding of DR pathogenesis and the characteristics of early-stage disease. Additionally, the neurovascular unit, which is associated with the main mechanism of DRN, is also important in the brain homeostasis and damages of the neurovascular unit are observed in stroke, small vessel disease, and dementia. Such diseases were reported to be associated with pRNFL damages^17–19^. After all, damages to the pRNFL in T2DM patients may reflect the impaired neurovascular coupling of systemic neurodegenerative disease besides the early stage of DR.

The mean pRNFL thickness in the control group tended to decrease over time, but it was not statistically significant. In the DM group, the mean pRNFL thickness decreased significantly over time, as did the thicknesses of the superior and inferior segments. The pRNFLs of the superior and inferior segments are thicker than those of the temporal and nasal segments, which would be associated with such patterns of pRNFL loss^10^. Additionally, this pattern of the pRNFL thinning is also observed in patients with open-angle glaucoma, which preferentially involves superior and inferior pRNFL losses^20,21^. DRN and open-angle glaucoma have common mechanisms such as glial dysfunction, retinal ganglion cell loss by cellular apoptosis, and impaired microcirculation, which results in pRNFL thinning. Therefore, such similar patterns and mechanisms of pRNFL loss would be helpful to explain the relationship between the 2 diseases that T2DM is one of the risk factors for glaucoma. In patients with DM duration ≥ 10 years, the mean pRNFL thicknesses and those of all four sectors showed a significant reduction over time. The nasal and temporal segments, which are less affected by other diseases, were nonetheless affected by DRN with extensive loss of pRNFL in prolonged T2DM.

The reduction rate of mean pRNFL in the control group was − 0.45 μm/year, which was consistent with previous studies^10,22–26^. Shin et al.^27^ found that the rate of change in mean pRNFL thickness was − 1.26 μm/year in patients with progressing glaucoma. Another study reported that the average RNFL thinning rate was − 1.31 μm/year in patients with pseudoexfoliative glaucoma^28^. Lee et al.^25^ reported that the reduction rate in the mean pRNFL thickness of the hypertension group was − 0.99 μm/year. The DM group exhibited a pRNFL thickness reduction rate similar to those of patients with other diseases associated with RNFL thinning, which was − 1.34 μm/year. In patients with DM duration ≥ 10 years, the reduction rate of mean pRNFL thickness was − 1.61 μm/year, which is significantly higher than those in the control group and the DM group. T2DM induces activation of microglial cells, the main sentinel immune cells located in the inner part of the retina, which then migrates to the subretinal space and releases cytokines contributing to neuronal cell death^7,29^. In prolonged T2DM, activated microglial cells would accumulate, triggering rapid DRN. Prolonged accumulation of glutamate and the loss of neuroprotective factors may also be associated with rapid pRNFL loss.

The BCVA was significantly associated with pRNFL changes in the DM group. Vujosevic et al.^9^ found significant correlations between pRNFL thickness and OCTA parameters such as perfusion or vessel densities in the parapapillary regions of patients with T2DM. Change in the peripapillary microvasculature is known to be associated with visual function decline in patients with various diseases including glaucoma^30–33^. Damages to the pRNFL affecting peripapillary microvasculature via the retinal neurovascular unit may thus affect visual acuity^7,11^. However, the BCVA was not significantly different between the two groups in our study, and Li et al.^34^ found no significant correlations between LogMAR BCVA and peripapillary capillary density in T2DM patients without DR. Although DRN may compromise various visual functions, further studies enrolling more cases are required to explore a possible direct relationship between DRN and visual acuity.

The HbA1c level significantly affected the pRNFL changes in the DM group. Zeng et al.^12^ reported that the implicit time for 16 and 32 Tds stimuli was positively associated with the level of HbA1c (r = 0.434, *P* = 0.024; r = 0.448, *P* < 0.001, respectively) in patients with T2DM without DR. The HbA1c level is a good indicator of blood glucose control in around 3 months, and long-term hyperglycemia could induce retinal hypoxia and inflammation, impairing retinal structures and neural function^35–37^. Therefore, good glycemic control is crucial to prevent or minimize the inner retinal damages by DRN. Meanwhile, Sohn et al.^38^ reported that the HbA1c level did not significantly influence the thickness of the RNFL in patients with type 1 DM in their longitudinal study. However, they analyzed the RNFL thickness of parafoveal and perifoveal areas. So RNFL thickness of the peripapillary area, which is much thicker than that of the macular area, may show the inner retinal damage caused by hyperglycemia more sensitively.

In patients with DM duration ≥ 10 years, hypertension significantly affected the changes in pRNFL thickness over time. Previous studies reported a significantly thinner pRNFL and rapid reduction of pRNFL in patients with hypertension^25,39,40^. Cantor et al.^41^ reported that glaucomatous damage could occur in hypertension patients because of ischemia in the optic nerve or retinal ganglion cell, which is related to microvascular pathologies such as atherosclerosis, increased resistance, rigidity, or insufficient autoregulation. When activated microglial cell numbers rise and glutamate accumulates in patients with prolonged T2DM, ischemic damages caused by hypertension may accelerate injury of the inner retina. However, further studies are needed with detailed histories of hypertension such as duration of disease and medication of hypertension.

Age did not significantly influence the changes in pRNFL thickness over time in patients with DM duration ≥ 10 years. Previous longitudinal studies reported that age was not a significant factor associated with changes in the pRNFL thickness of T2DM patients^10,38^. Lee et al.^42^ also reported that age was not a significant factor associated with retinal microvasculature in T2DM. These studies all reported that DM duration was a significant factor associated with the inner retinal damage by DRN and impairment of the retinal microvasculature. However, although the results of the above studies showed that the DM duration is more important, age was a significant factor affecting the pRNFL change in the DM group in this study. Furthermore, age is well known to play a key role in the etiology of the neurodegenerative diseases, such as Alzheimer’s disease and Parkinson’s disease, by the increased burden of systemic, chronic, and low-grade inflammatory condition, characterized by persistent and non-resolved production of pro-inflammatory mediators^43–45^. Therefore, age should not be overlooked as a factor affecting neurodegeneration including DRN.

The major limitation of our study was that we did not perform the various visual function test such as visual field test, contrast test, color vision test, or electroretinography so we could not analyze the association between various visual functions and DRN. Additionally, much more data about blood glucose level or blood pressure through more patient visits would allow more detailed analyses of any effect of hyperglycemia and hypertension. Third, the control group may not represent the real healthy population as most of them came to the retina clinic regularly for their fellow eyes with a disease, which may introduce a selection bias. The strength of our study is that this is the first study to identify longitudinal changes in pRNFL thickness with analyses for patients with DM duration ≥ 10 years for the detailed analysis of how the T2DM duration affects DRN.

In conclusion, we confirmed rapid pRNFL thickness reduction over time in patients with T2DM, and the reduction rate was higher in patients with T2DM ≥ 10 years. In patients with T2DM, pRNFL reductions were evident principally in the superior and inferior segments, which was a similar pattern with glaucoma patients. In patients with T2DM ≥ 10 years, significant reductions were observed in all four segments. Additionally, the BCVA and HbA1c level were significantly associated with changes in pRNFL thickness over time in patients with T2DM, and hypertension was a significant factor affecting the change of pRNFL in patients with T2DM ≥ 10 years. Physicians should be aware that severe and rapid DRN may develop in patients with prolonged T2DM and consider these findings when interpreting pRNFL changes in patients with T2DM.

## Supplementary information

Supplementary table.
