# Supplementary material for: Effects of prolonged type 2 diabetes on changes in peripapillary retinal nerve fiber layer thickness in diabetic eyes without clinical diabetic retinopathy
Source: Sci Rep. 2021 Mar 24;11:6813. doi: 10.1038/s41598-021-86306-y (PMC7991659; doi:10.1038/s41598-021-86306-y)
Supplement: Supplementary file 1 — Supplementary table. [file 41598_2021_86306_MOESM1_ESM.pdf]

Supplementary table. Peripapillary retinal nerve fiber layer thickness in each group

	Control	DM group	P-value*
Mean			
Baseline	95.8 ± 8.1	93.4 ± 9.1	0.102
First year	95.4 ± 8.3	92.1 ± 9.3	**0.031**
Second year	94.9 ± 8.1	90.9 ± 9.3	**0.010**
Third year	94.5 ± 8.3	89.5 ± 9.2	**0.008**
P-value†	0.138	**< 0.001**	
Superior segment			
Baseline	120.2 ± 14.0	116.1 ± 16.2	0.128
First year	118.6 ± 14.9	114.2 ± 16.0	0.112
Second year	118.4 ± 13.4	112.4 ± 17.6	**0.016**
Third year	119.4 ± 13.7	110.4 ± 17.3	**0.010**
P-value†	0.110	**< 0.001**	
Temporal segment			
Baseline	70.3 ± 10.0	69.2 ± 13.9	0.604
First year	71.1 ± 10.6	69.5 ± 13.5	0.456
Second year	70.1 ± 10.1	68.3 ± 13.4	0.406
Third year	68.2 ± 8.9	67.6 ± 12.9	**0.010**
P-value†	0.151	0.130	
Inferior segment			
Baseline	123.7 ± 14.2	119.8 ± 14.9	0.129
First year	123.3 ± 16.1	119.2 ± 15.5	0.134
Second year	121.3 ± 16.1	117.3 ± 15.1	0.139
Third year	122.5 ± 15.8	115.3 ± 14.9	**0.024**
P-value†	0.095	**< 0.001**	
Nasal segment			
Baseline	67.5 ± 11.7	66.9 ± 6.6	0.694
First year	67.1 ± 11.0	66.0 ± 6.9	0.496
Second year	66.9 ± 10.8	64.8 ± 6.5	0.159
Third year	67.5 ± 10.5	63.6 ± 7.1	0.075
P-value†	0.671	0.065	

*Calculated for the independent t-test.
†Calculated for repeated-measures ANOVA.
Values in boldface (P < 0.05) are statistically significant.
All values are expressed as the mean ± standard deviation (μm).
